# Adjuvant radiotherapy shows benefit in selected stage I uterine sarcoma: A risk scoring system based on a population analysis

**DOI:** 10.1002/cam4.4643

**Published:** 2022-03-11

**Authors:** Yun‐xia Huang, Yan‐zong Lin, Yi‐min Li, Ke‐xin Chu, Yu‐fei Zhou, Li‐mei Lin, Rui Zhou, Zong‐kai Zhang, Qin Lin

**Affiliations:** ^1^ Department of Radiation Oncology, The First Affiliated Hospital of Xiamen University, School of Medicine, Xiamen University Teaching Hospital of Fujian Medical University Xiamen China; ^2^ Department of General Surgery, The First Affiliated Hospital of Xiamen University, School of Medicine, Xiamen University Teaching Hospital of Fujian Medical University Xiamen China

**Keywords:** adjuvant radiotherapy, prognosis, SEER, stage I uterine sarcoma

## Abstract

**Background:**

The potential therapeutic benefit of adjuvant radiotherapy for patients with stage I uterine sarcoma has not been clear. In this study, we aimed to develop a risk scoring model to select the subgroup of patients with stage I uterine sarcoma who might benefit from adjuvant radiotherapy.

**Methods:**

Patients with stage I uterine sarcoma from the Surveillance, Epidemiology, and End Results program from 2010 to 2014 were retrospectively included in this analysis. Cox proportional hazards models were performed to identify risk factors.

**Results:**

A total of 947 stage I uterine sarcoma patients were included. The 5‐year disease‐specific survival (DSS) of the overall cohort was 75.81%. Multivariate analysis identified stage (*p* = 0.013), tumor grade (*p* <0.001) and histology (*p* = 0.043) as independent prognostic factors for DSS, and these factors were used to generate the risk scoring model. The low‐risk group presented a better DSS than the high‐risk group (95.51% vs. 49.88%, *p* < 0.001). The addition of radiotherapy to surgery significantly increased the DSS in the high‐risk group compared with surgery alone (78.06% vs. 46.88%, *p* = 0.022), but no significant survival benefit was observed in the low‐risk group (98.36% vs. 100%, *p* = 0.766).

**Conclusions:**

Our risk scoring model based on stage, tumor grade, and histology predicted the outcome of patients with stage I uterine sarcoma cancer. This system may help to select stage I uterine sarcoma cancer patients who might benefit from adjuvant radiotherapy.

## Graphical abstract

Our study defines the risk factors for the disease‐specific survival (DSS) of patients with stage I uterine sarcoma and develops a risk‐scoring model that would be able to select the subgroup who might benefit from adjuvant radiotherapy.

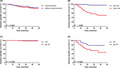

## 1 INTRODUCTION

Uterine sarcomas are rare tumors that originate from the myometrium and connective tissue elements, comprising 3% to 7% of all uterine malignancies, and 4910 cases were estimated in 2017 in the United States.^1^, ^2^ The World Health Organization (WHO) 2003 classification broadly divides uterine sarcomas into two groups: mesenchymal tumors, which include leiomyosarcomas (LMS, 63%), endometrial stromal sarcomas (ESS, 21%) and other types (10%); and mixed epithelial‐mesenchymal tumors, which include adenosarcomas (AS, 6%).^3^, ^4^

LMS are very aggressive tumors that are characterized by severe nuclear atypia and a high mitotic rate. Patients with LMS at stage I show a poor 5‐year survival of 51%.^5^, ^6^ ESS are regarded as indolent tumors, and stage I–II patients show a favorable 5‐year disease‐specific survival (DSS) of 89.3%.^7^ Most AS present mild to moderate nuclear atypia in the stromal component, and patients with stage I AS show a favorable 5‐year survival of approximately 76%.^6^, ^8^

The standard treatment for stage I uterine sarcoma is total hysterectomy with or without bilateral salpingo‐oophorectomy (BSO).^9^ Several retrospective studies observed a trend of improved local control in early‐stage uterine sarcoma treated with postoperative radiotherapy (RT); however, this treatment produced no benefit on overall survival.^10^, ^11^ The only randomized data that reported no improvement in local control or survival with the addition of RT was performed in patients with stage I/II LMS.^12^ Stage I uterine sarcoma patients may be stratified into different prognostic groups and the treatment strategy should be tailored to each group.^13^ Thus, adjuvant RT may need to be individualized based on risk factors.

In this study, we evaluated the efficacy of postoperative RT for stage I uterine sarcoma using data extracted from Surveillance, Epidemiology, and End Results (SEER) and developed a risk scoring system.

## 2 MATERIALS AND METHODS

### 2.1 Patients

The SEER dataset is a comprehensive source of data for cancer incidence, prevalence, and mortality statistics in the United States and covers approximately 28% of the population in the USA.^14^ The SEER data are publicly available, and therefore institutional review approval was not required for this study.

A total of 2113 cases of uterine sarcoma (C540–549, 559, Uterus, NOS) with limitation to “one primary only” and “positive histology” between 2010 and 2014 were extracted from the SEER dataset (SEER*Stat 8.3.6). The histological types (ICD‐O‐3 codes) were limited to the following: LMS, 8890/3, 8891/3 and 8896/3; ESS, 8805/3, 8930/3 and 8931/3; and AS, 8933/3. Exclusion criteria were as follows: patients with stage II–IV disease (*n* = 1011); surgery type indicated as “no surgery performed,” “local excision,” or “subtotal hysterectomy” (*n* = 111); and survival time listed as “0” (*n* = 44). Finally, 947 patients with stage I uterine sarcoma who underwent total hysterectomy were included in our study. And tumor grade or stage record as “unknown” (*n* = 430) were excluded before performing Cox analysis.

The AJCC 7th Edition TNM staging reflects the new staging adopted by the International Federation of Gynecology and Obstetrics in SEER program. The disease extent according to SEER is as follows. Localized stage (T1N0M0, stage I) is defined as the tumor limited to the uterus, which corresponds to FIGO stage I. For LMS and ESS, sub‐classification into FIGO stage IA and IB disease was based on tumor size (<5 cm, ≥5 cm). For AS, tumors limited to the endometrium/endocervix were defined as stage IA, while tumors that invaded to <1/2 and ≥1/2 of the myometrium were defined as stage IB and IC, respectively.

### 2.2 Statistical analysis

The study endpoint was DSS, which was defined as the duration of time between the date of diagnosis to cancer‐related death or last follow‐up. Comparison of demographic and tumor characteristics between two treatment groups was performed by chi‐square (χ2) test. Kaplan–Meier method was used to estimate the DSS. Cox regression model was performed to identify risk factors. Risk scores were obtained from the β regression coefficient. The cut‐off point impacting DSS was determined by the receiver operating characteristic curve (ROC). Statistical analyses were performed using SPSS version 22.0 (IBM Corporation). A *p*‐value <0.05 was considered statistically significant.

## 3 RESULTS

### 3.1 Patient characteristics

**FIGURE 1:**
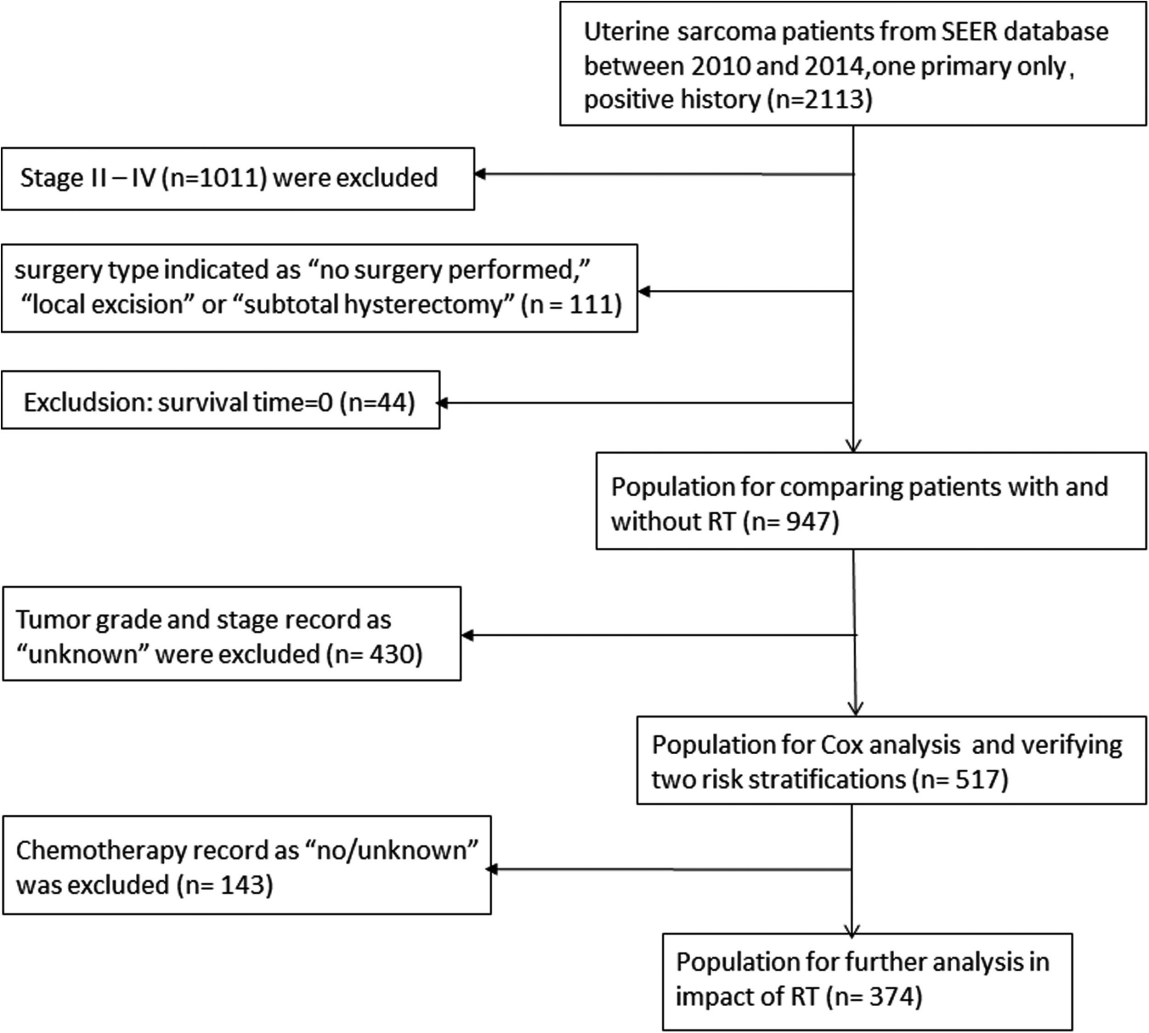
Flowchart of the study design

**FIGURE 2:**
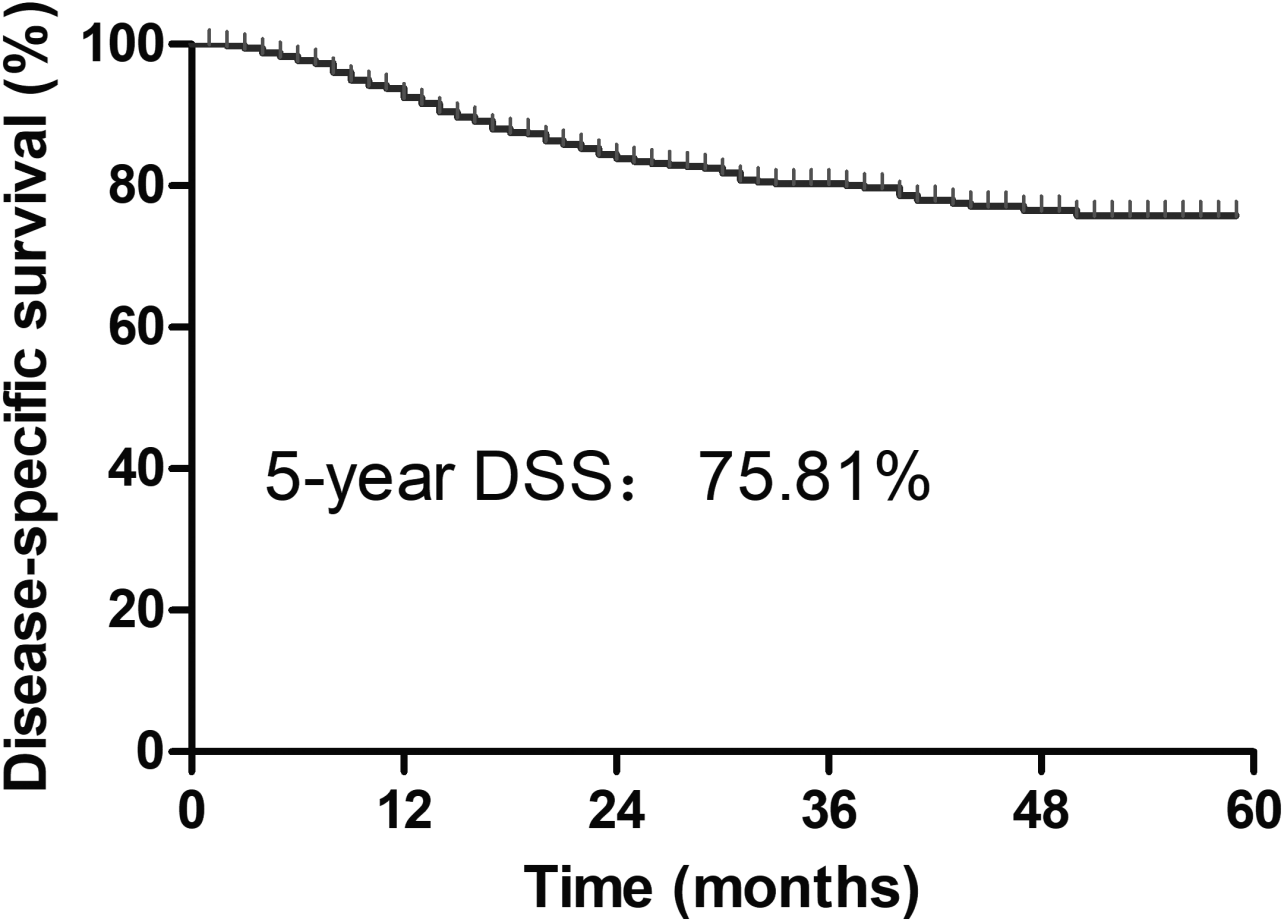
Overall survival of 947 stage I uterine sarcoma patients

A total of 947 stage I uterine sarcoma patients were retrospectively analyzed, and Figure 1 depicts the flow chart of the study. The study group included 829 patients who received surgery alone and 118 patients who received surgery combined with postoperative RT. All patients underwent standard surgery with a total hysterectomy. The median follow‐up time was 53 months (24–82 months), and the 5‐year DSS of the overall cohort was 75.81% (95% confidence interval [CI]: 71.50%–79.57%) (Figure 2).

The demographics and characteristics of the patients with and without RT included in this study are listed in Table 1. Postoperative RT was more common in patients with medical insurance than those without (*p* = 0.004), and postoperative RT was more common among patients >50 years old compared with patients ≤50 years old (*p* = 0.001). Patients with stage IB or IC more commonly received RT than patients with stage IA (*p* = 0.036). Patients with grade III/IV more commonly received RT than patients with grade I/II (*p* <0.001). Postoperative RT was more common in patients who received chemotherapy than those who did not (*p* <0.001).

**TABLE 1:** Demographic and tumor characteristics of uterine sarcoma patients with and without RT

Characteristic	Total, *n* (%)	S, *n* (%)	S + RT, *n* (%)	*p*
	947 (100)	829 (87.54)	118 (12.46)	
Race				0.658
White	680 (71.81)	600 (72.4)	80 (67.8)	
Black	160 (16.90)	136 (16.4)	24 (20.3)	
Other	105 (11.09)	91 (11)	14 (11.9)	
Unknown	2 (0.2)	2 (0.2)	0 (0)	
Marital status				0.292
Married	500 (52.80)	432 (52.1)	68 (57.6)	
Single/Unmarried	217 (22.91)	190 (22.9)	27 (22.9)	
Divorced/Widowed	170 (17.95)	150 (18.1)	20 (16.9)	
Unknown	60 (6.34)	57 (6.9)	3 (2.5)	
Insurance				0.004
Yes	880 (92.93)	776 (93.6)	104 (88.1)	
No	54 (5.70)	40 (4.8)	14 (11.9)	
Unknown	13 (1.37)	13 (1.6)	0 (0)	
Age				0.001
≤50 years	393 (41.50)	360 (43.4)	33 (28)	
>50 years	554 (58.50)	469 (56.6)	85 (72)	
Histology				0.871
LMS	507 (53.54)	443 (53.4)	64 (54.2)	
AS & ESS	440 (46.46)	386 (46.6)	54 (45.8)	
Stage				0.036
Stage IA	280 (29.57)	253 (30.5)	27 (22.9)	
Stage IB & IC	547 (57.76)	466 (56.2)	81 (68.6)	
Unknown	120 (12.67)	110 (13.3)	10 (8.5)	
Tumor grade				<0.001
Grade I/II	283 (29.88)	269 (32.4)	14 (11.9)	
Grade III/IV	298 (31.47)	234 (28.2)	64 (54.2)	
Unknown	366 (38.65)	326 (39.3)	40 (33.9)	
Tumor size, median (cm)	7.9	7.6	8.0	
Surgery				0.617
Oophorectomy	730 (77.09)	639 (77.1)	91 (77.1)	
No oophorectomy	139 (14.68)	124 (15.0)	15 (12.7)	
Unknown	78 (8.24)	66 (8.0)	12 (10.2)	
Chemotherapy				<0.001
Yes	699 (73.81)	628 (75.8)	71 (60.2)	
No	248 (26.19)	201 (24.2)	47 (39.8)	

Abbreviation: S, surgery alone; S + RT, surgery combined with adjuvant radiotherapy.

### 3.2 Subgroup analyses

**FIGURE 3:**
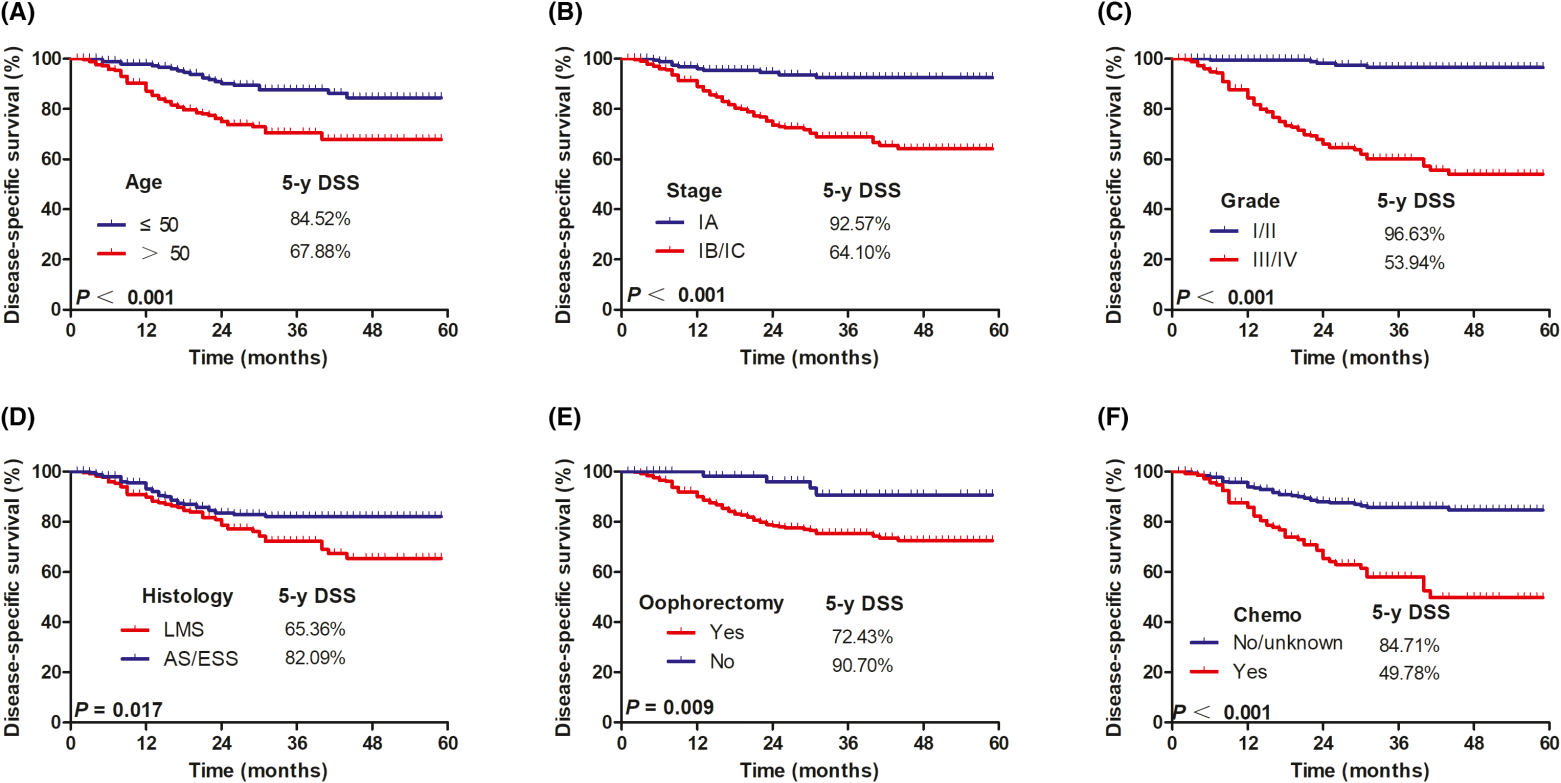
Kaplan–Meier analysis of disease‐specific survival according to (A) age (≤50 vs. >50 years, *p <* 0.001); (B) stage (IA vs. IB/IC, *p <*0.001); (C) tumor grade (grade I/II vs. grade III/IV, *p <*0.001); (D) histology (LMS vs. AS/ESS, *p =* 0.017); (E) oophorectomy (Yes vs. No, *p =* 0.009); (F) chemotherapy (Yes vs. No, *p <*0.001)

We observed a more favorable outcome in patients ≤50 years old than those >50 years old (DSS: 84.52% vs. 67.88%, *p* <0.001, Figure 3A), and patients with stage IA had a more favorable outcome than patients with stage IB or IC (DSS: 92.57% vs. 64.10%, *p* <0.001, Figure 3B). A better outcome was observed in patients with grade I/II cancer than those with grade III/IV cancer (DSS: 96.63% vs. 53.94%, *p* <0.001, Figure 3C).

**FIGURE 4:**
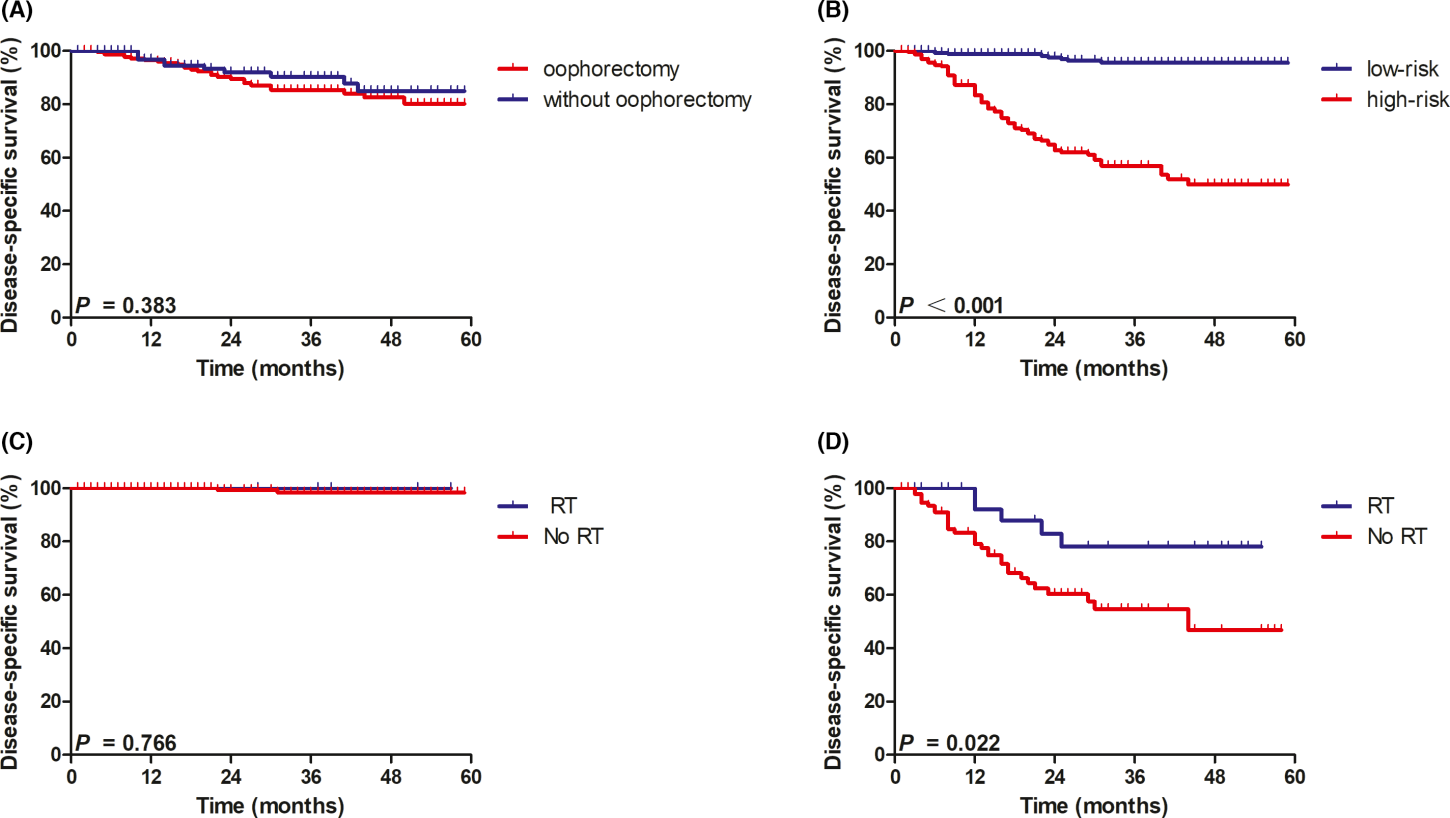
Kaplan–Meier analysis of disease‐specific survival according to (A) oophorectomy for patients age ≤ 50 years old (Yes vs. No, *p =* 0.383); (B) risk stratifications (low risk vs. high risk, *p* <0.001); (C) RT for low‐risk patients (Yes vs. No, *p* = 0.766); and (D) RT for high‐risk patients (Yes vs. No, *p* = 0.022)

Subgroup analysis according to histological subtype revealed a better DSS in patients with AS/ESS (82.09%) compared with patients with LMS (65.36%) (*p* = 0.017, Figure 3D). A better prognosis was observed in patients with preserved ovaries compared with patients who received oophorectomy (DSS: 90.70% vs. 72.43%, *p* = 0.009, Figure 3E). The addition of chemotherapy was also associated with a worse outcome (with chemotherapy vs. without chemotherapy, DSS: 49.78% vs. 84.71%, *p* <0.001, Figure 3F). Subgroup analysis for premenopausal women (age ≤ 50 years old) with stage I uterine sarcoma revealed no survival benefit for patients who received oophorectomy (80.17%) compared with patients who received oophorectomy (84.96%) (*p* = 0.383, Figure 4A).

### 3.3 Cox analysis for DSS

Univariate analyses revealed that age (*p* <0.001), stage (*p* <0.001), tumor grade (*p* <0.001), histology (*p* = 0.017), surgery (*p* = 0.009), and chemotherapy (*p* <0.001) were associated with DSS (Table 2). Furthermore, multivariate analyses showed that stage (hazard ratio [HR]: 2.487, 95% CI: 1.211–5.108, *p* = 0.013), tumor grade (HR: 12.632, 95% CI: 4.814–33.148, *p* <0.001), and histology (HR: 1.618, 95% CI: 1.036–2.632, *p* = 0.043) were independent prognostic factors for DSS.

**TABLE 2:** Univariate and multivariate analyses for 517 uterine sarcoma patients

Variable	Univariate analysis		Multivariate analysis	
	HR (95% CI)	*P*	HR (95% CI)	*P*
Race				
White	Reference	0.106		
Black	2.177 (0.876–5.411)	0.094		
Other	2.924 (1.079–7.925)	0.035		
Marital status				
Married	Reference	0.660		
Single/Unmarried	0.788 (0.471–1.317)	0.363		
Divorced/Widowed	0.837 (0.444–1.577)	0.582		
Insurance				
Yes/No	0.887 (0.325–2.324)	0.815		
Age, years				
≤ 50 vs. >50	2.728 (1.651–4.508)	<0.001	1.300 (0.751–2.249)	0.349
Stage				
IA vs. IB/IC	5.013 (2.589–9.706)	<0.001	2.487 (1.211–5.108)	0.013
Grade				
I/II vs. III/IV	18.712(7.571–46.243)	<0.001	12.632 (4.814–33.148)	<0.001
Histology				
LMS/AS & ESS	0.592 (0.385–0.910)	0.017	1.618 (1.036–2.632)	0.043
Oophorectomy				
Yes/No	0.261 (0.095–0.714)	0.009	0.511 (0.179–1.461)	0.210
Radiotherapy				
Yes vs. No	1.527 (0.908–2.570)	0.111		
Chemotherapy				
Yes vs. No	3.487 (2.271–5.355)	<0.001	1.445 (0.909–2.297)	0.120

*Note*. Unknown data points were removed before performing statistical tests.

### 3.4 Development of the risk scoring model

As shown in Table 3, after weighting each risk factor by the beta regression coefficient and Exp (B), 14 points, 2 points, and 2 points were assigned to grade III/IV tumor, LMS, and stage IB or IC, respectively (Table 3). We used the scoring system to calculate the total score for each patient by the addition of the above three risk factors. ROC identified two risk stratifications: 276 cases (53.4%) with a low risk of cancer death (score < 14) and 241 (46.6%) cases with a high risk of cancer death (score ≥ 14).

**TABLE 3:** Risk variables for the scoring system

Risk variable	Sig.	Exp(B)	Risk coefficient	Risk score
Grade	<0.001	12.632		
I/II			0	0
III/IV			12.632	13
Histology	0.043	1.618		
AS/ESS			0	0
LMS			1.618	2
Stage	0.013	2.487		
IA			0	0
IB & IC			2.487	2

### 3.5 Survival analysis based on the risk scoring model

A significant difference in survival was observed between the low‐risk and high‐risk groups (95.51% vs. 49.88%, *p* <0.001; Figure 4B). We further analyzed the impact of RT on outcome and patients who received chemotherapy were excluded. In the low‐risk group, patients did not significantly benefit from the addition of RT (98.36% vs. 100%, *p* = 0.766; Figure 4C). However, among high‐risk patients, a significantly better outcome was found in patients with RT (78.06% vs. 46.88%, *p* = 0.022; Figure 4D, Table 4).

**TABLE 4:** Survival analysis of two risk stratifications

Risk stratification	Without radiotherapy		With radiotherapy		*p*
	*n* (%)	5‐year DSS (%)	*n* (%)	5‐year DSS (%)	
Low‐risk group	235 (94.5)	98.36	11 (4.48)	100	0.766
High‐risk group	97 (75.8)	46.88	31 (24.2)	78.06	0.022

Abbreviation: DSS, disease‐specific survival.

## 4 DISCUSSION

Surgery is the principal and mainstay treatment for stage I uterine sarcomas.^9^ Adjuvant RT is generally not performed for stage I uterine sarcoma patients in clinical practice. However, the use of RT remains somewhat controversial, as several retrospective reviews have shown convincing evidence supporting the use of RT for patients with stage I uterine sarcoma. Thus, identifying patients that might benefit from adjuvant RT is critical. Here, we developed a risk‐scoring model including easily available clinical and pathological factors to identify the subset of stage I uterine sarcoma patients who will benefit from RT. Our results identified tumor grade, histology, and stage as independent prognostic factors for stage I uterine sarcomas. We combined the three risk factors into a scoring model and further classified patients into two risk groups. A significantly favorable outcome was found for high‐risk patients by the addition of RT (78.06% vs. 46.88%, *p* = 0.022), while no significant benefit from RT was observed among low‐risk patients (98.36% vs. 100%, *p* = 0.766).

The role of adjuvant therapy for surgically treated stage I uterine sarcomas has been unclear. A phase III randomized study (EORTC 55874) indicated that postoperative RT showed no benefit in local control or overall survival for stage I–II uterine sarcoma patients.^12^ However, 41.1% of patients in the EORTC study had carcinosarcoma, which has not been categorized as a uterine sarcoma since the mid‐2000s. Therefore, subgroup analysis was not performed because of the limited sample numbers. In our study, no survival difference was observed between patients who received RT and those who did not in the total population. RT should be tailored to the selected high‐risk groups. Our study indicated that adjuvant RT was not recommended for uterine sarcoma patients with grade I/II, regardless of histological type and stage. However, our results revealed that grade III/IV patients with LMS and/or stage IB/IC may benefit from adjuvant RT.

In this study, grade III/IV, stage IB/IC, and LMS were identified as adversely independent prognostic factors for stage I patients with uterine sarcoma. Among the three factors, tumor grade was the strongest prognostic factor. Stage was the most consistent prognostic factor for survival, and this has been previously demonstrated in several studies.^15^, ^16^ Among the histological types of uterine sarcoma, LMS is aggressive, with a reported 5‐year survival of 51%–76% for stage I disease,^6^, ^17^ which is similar to the 65.36% rate in our study cohort. A previous study indicated that ESS and AS showed a more favorable 5‐year crude survival of 84% and 76%, respectively.^6^ Consistent with these data, the 5‐year DSS for ESS/AS in our study was 82.09%. Low‐grade ESS has the best survival prognosis among all histological subtypes of uterine carcinoma.^15^

The standard treatment recommended for uterine sarcoma is total hysterectomy with or without BSO.^9^ The aim of BSO is to eliminate the possibility of tumor recurrence from stimulation from endogenous steroid hormones, especially with ESS or tumors expressing estrogen or progesterone receptors.^18^, ^19^ In our study, the 5‐year DSS rates for patients who underwent oophorectomy (*n* = 401) and those who did not undergo oophorectomy (*n* = 68) were 72.43% and 90.70%, respectively. However, several retrospective studies found that oophorectomy was not associated with better survival for premenopausal women with stage I uterine sarcoma.^20^, ^21^ Similarly, our subgroup analysis revealed no survival difference for patients with or without oophorectomy for stage I uterine sarcoma patients age ≤ 50 years old. Patients over 50 years of age with later disease stage and worse tumor grade were significantly more likely to undergo oophorectomy. There were no differences between the two groups in terms of histology or patient race. Ovarian preservation is safe and should be encouraged for premenopausal women with stage I uterine sarcoma.

The role of adjuvant chemotherapy for patients with stage I uterine sarcoma is also poorly defined. A prospective trial in 1995 found no long‐term survival benefit of adjuvant chemotherapy (cyclophosphamide, vincristine, doxorubicin, and dacarbazine) for stage I uterine sarcoma.^22^ Given the high risk of distant recurrence, NCCN guidelines currently recommend that systemic therapy should be considered for stage I LMS and high‐grade ESS (category 2B).^23^ A phase III randomized trial (GOG 277) evaluating the benefit of adjuvant chemotherapy versus observation in high‐grade stage I and II LMS is underway.^24^ Univariate analysis in our study showed that the addition of chemotherapy was associated with worse survival, which may be because patients receiving chemotherapy presented with several risk factors (age > 50, grade III/IV, stage IB/IC, and histology with LMS), which adversely affected survival.

This study had several limitations. First, the SEER dataset lacked data on local control, which is a vital outcome that reflects the effect of RT. Second, there were no details on chemotherapy administration and the RT regimen, which are potential confounders in our study. Third, increasing research has reported the predictive value of molecular biomarkers for uterine sarcoma; however, our model was unable to integrate molecular information, as this information was not available in the SEER database. Additionally, no external data were available to verify our conclusions.

## 5 CONCLUSION

In our study, we identified stage, tumor grade, and histology as significant prognostic variables for stage I uterine sarcoma based on analysis of SEER program. Our risk scoring model based on these clinical factors may help identify patients with stage I uterine sarcoma who can benefit from RT.

## CONFLICT OF INTEREST

The authors report no conflicts of interest in this work.

## AUTHORS' CONTRIBUTIONS

All authors helped to perform the research. Yun‐xia Huang and Yan‐zong Lin carried out manuscript writing and performing procedures, Yi‐min Li, Ke‐xin Chu, and Yu‐fei Zhou were involved in manuscript writing and data analysis. Li‐mei Lin, Rui‐zhou, Zong‐kai Zhang, and Qin Lin contributed to drafting conception and design of the manuscript. All authors approved the final manuscript.

## ETHICAL APPROVAL STATEMENT

The SEER data are publicly available, and therefore institutional review approval was not required for this study.

## Data Availability

The data that support the findings of this study are available in the Surveillance, Epidemiology, and End Results (SEER) program. These data were derived from the following resources available in the public domain: https://seer.cancer.gov.
